# O-linked β-N-acetylglucosamine transferase plays an essential role in heart development through regulating angiopoietin-1

**DOI:** 10.1371/journal.pgen.1008730

**Published:** 2020-04-06

**Authors:** Yongxin Mu, Houzhi Yu, Tongbin Wu, Jianlin Zhang, Sylvia M. Evans, Ju Chen

**Affiliations:** 1 Department of Medicine-Cardiology, University of California San Diego,Gilman Drive, Mail Code, La Jolla, California, United States of America; 2 Department of Cardiology, Shandong Provincial Hospital affiliated to Shandong University, Jinan, China; 3 Skaggs School of Pharmacy and Pharmaceutical Sciences, University of California at San Diego, La Jolla, CA, United States of America; Indiana University Purdue University at Indianapolis, UNITED STATES

## Abstract

O-linked N-acetylglucosamine (GlcNAc) transferase (OGT) is the only enzyme catalyzing O-GlcNAcylation. Although it has been shown that OGT plays an essential role in maintaining postnatal heart function, its role in heart development remains unknown. Here we showed that loss of OGT in early fetal cardiomyocytes led to multiple heart developmental defects including hypertrabeculation, biventricular dilation, atrial septal defects, ventricular septal defects, and defects in coronary vessel development. In addition, RNA sequencing revealed that Angiopoietin-1, required within cardiomyocytes for both myocardial and coronary vessel development, was dramatically downregulated in cardiomyocyte-specific OGT knockout mouse hearts. In conclusion, our data demonstrated that OGT plays an essential role in regulating heart development through activating expression of cardiomyocyte Angiopoietin-1.

## Author summary

As the first functional organ to form, the heart develops from a simple tube to a complex organ with four chambers to keep up with the body’s increasing demand for blood. During this period, myocardium, the major component of heart, undergoes substantial dynamic architectural remodeling at different levels. This remodeling also coincides with coronary vessel development to form a functional heart. Defects in architectural remodeling of myocardium or coronary vessel development can lead to anatomical changes during heart development, resulting in congenital heart disease. O-linked N-acetylglucosamine (GlcNAc) transferase (OGT) is the only enzyme catalyzing protein O-GlcNAcylation using UDP-GlcNAc, the end product of the hexosamine biosynthetic pathway. Hence, OGT is also considered a sensor of cellular nutrient levels. In this study, we found that loss of OGT in myocardium disrupted myocardial remodeling, as well as coronary vessel development, through regulating Angiopoietin-1 expression. Our study not only demonstrates an essential role for myocardial OGT during heart development, but also has potential implications for the development of new treatment options targeting coronary diseases.

## Introduction

O-GlcNAcylation is a post-translational modification that occurs by the addition of single O-linked β-N-acetylglucosamine (O-GlcNAc) moieties to serine or threonine residues, in a manner analogous to that of protein phosphorylation. In contrast to the existence of hundreds of kinases and phosphatases, O-GlcNAc transferase (OGT) and O-GlcNAcase (OGA) are the only enzymes responsible for addition and removal of O-GlcNAc respectively [1, 2]. Since O-GlcNAcylation was first reported, more than one thousand proteins have been shown to be O-GlcNAcylated. O-GlcNAcylation affects diverse cellular and molecular processes, including protein-protein interactions, transcriptional regulation, autophagy, cell proliferation and apoptosis, and insulin sensitivity [3–5].

Since the heart shows relatively high levels of OGT activity, and a number of essential cardiac proteins are O-GlcNAcylated, the function of O-GlcNAc in adult heart has been extensively studied [6, 7]. Global increases in O-GlcNAcylation have been reported in heart samples of patients with aortic stenosis, and similar observations have been made in rat models of heart failure induced by hypertension, myocardial infarction, or aortic constriction [8]. Data from both in vitro and in vivo models suggest that increased O-GlcNAcylation plays a cardioprotective role in settings of acute cardiac dysfunction, such as ischemia-reperfusion and trauma-hemorrhage, while O-GlcNAcylation may show deleterious effects on cardiac function in chronic conditions such as diabetic cardiomyopathy [6]. Deletion of OGT in adult mouse heart accelerates the progression of ischemia-induced heart failure without affecting cardiomyocyte size [9]. In addition, constitutive deletion of cardiomyocyte OGT causes partial perinatal lethality, and surviving knockout mice showed heart failure with more fibrosis, apoptotic cells and hypertrophy [10]. However, the role of OGT and O-GlcNAcylation during early heart development remains unclear.

Angiopoietin-1 (Angpt1) is the first identified member of the angiopoietin family and the major ligand for Tie2, an essential tyrosine kinase largely restricted to endothelial cells [11–13]. As one of the major communication signaling pathways between endothelial cells and vascular support cells, Angpt1/Tie2 signaling is required for the maintenance of endothelial cell quiescence, pericyte recruitment and maturation of vessels [14]. Cooperating with VEGF signaling, Angpt/Tie2 signaling controls sprouting angiogenesis, vascular remodeling and transition from quiescent to activated endothelial cells [11, 12]. Both Angpt1 global and cardiac specific knockout mice exhibit embryonic lethality with dramatic anomalies in heart trabeculation [13, 15, 16]. Cardiomyocyte specific Angpt1 knockout embryos also show severe defects in coronary vessel development [15]. Although cardiomyocyte Angpt1 is essential for heart development, the regulation of Angpt1 expression in cardiomyocytes is still largely unknown.

In this study, we found that loss of OGT in early fetal cardiomyocytes causes dilated biventricular non-compaction together with multiple heart developmental defects. Development of coronary vessels, in particular coronary arteries, is also affected by loss of cardiomyocyte OGT. RNA sequencing revealed that Anpgt1 was dramatically downregulated in cardiomyocyte specific OGT knockout mouse hearts, consistent with heart developmental defects observed consequent to loss of OGT. In conclusion, our data shows that OGT plays an essential role in heart development, in part through regulating Angpt1 expression.

## Materials and methods

### Ethics statement

All animal procedures were performed in accordance with the National Institutes of Health Guide for the Care and Use of Laboratory Animals and approved by the Institutional Animal Care and Use Committee of the University of California, San Diego with approved protocol # S01049.

### Animal models

*Ogt*^flox/flox(f/f)^ mouse was purchased from Jackson Lab (Stock No:004860 | OGTF). To generate cardiomyocyte-specific OGT KO mice, floxed mice were crossed with cardiac *Troponin T* (*TnT)-Cre* transgenic mice [17] to create *Ogt*^f/y^; *TnT-Cre* (cKO) mice. All mice were of a mixed 129/SvJ and C57BL/6J background. NKX2-5 knockout mouse was described in previous report [18]. Genotypes of mice were confirmed by polymerase chain reaction (PCR) analysis using embryonic yolk sac or tail extracts and *Ogt* primers (forward: 5’-CATCTCTCCAGCCCCACAAACTG-3’, reverse: 5’-GACGAAGCAGGAGGGGAGAGCAC-3’), *Cre* primers (forward: 5’-GTTCGCAAGAACCTGATGGACA-3’; reverse: 5’-CTAGAGCCTGTTTTGCACGTTC-3’) and *NKX2-5* primers (forward: 5’- CGGCATAGGACCAGAGTGATA-3’, reverse: 5’- TCCCTGAACATGTCCATCAGGTTC-3’).

### Histology

Histology was performed as previously described[19]. Briefly, mouse embryos were harvested at different stages and fixed in ice-cold phosphate buffered saline (PBS) containing 4% paraformaldehyde overnight at 4°C. Following fixation, embryos were washed with PBS, dehydrated with a series of 50–100% ethanol, embedded in paraffin, and cut into 8-μm sections using a Microtome. Sections were stained with Hematoxylin and Eosin (H&E), mounted, and imaged using a NanoZoomer 2.0HT Slide Scanning System (Hamamatsu).

### Protein Isolation, Western Blot Analyses and associated antibodies

Protein isolation and western blot analysis were performed as described in our previous publication [19]. Briefly, embryo hearts were harvested and snap-frozen in liquid nitrogen. Frozen heart tissue was homogenized in 80 μL RIPA buffer (50 mM Tris⋅Cl, pH 7.4, 150 mM sodium chloride, 1% Nonidet P-40, 0.1% SDS, 0.5% sodium deoxycholate, 1 mM EDTA) using a handheld pestle (Sigma-Aldrich). Protein concentration was determined using a Micro BCA Protein Assay Kit (Thermo Fisher Scientific) before mixing with 4× lithium dodecyl sulfate (LDS) sample buffer (Life Technologies) and incubating for 10 min at 70°C. Proteins were separated on 4–12% SDS-PAGE gels (Life Technologies) and transferred on to nitrocellulose membranes (Biorad) at 4°C at a constant voltage of 35V in transfer buffer (25 mmol/L Tris, 190 mmol/L glycine, 20% methanol, pH 8.3). After blocking for 1–2 hours in blocking buffer (Tris buffered saline containing 0.1% Tween 20 and 5% non-fat dry milk), membranes were incubated overnight at 4°C with the indicated primary antibody in blocking buffer. Blots were washed and incubated with horseradish peroxidase–conjugated secondary antibody for 1 hour at room temperature. Immunoreactive protein bands were visualized using enhanced chemiluminescence reagent (Thermo), analyzed by densitometry, and expressed as pixel density normalized to GAPDH. Primary antibodies include: OGT (1:1000, 61355, Active Motif), RL2 (1:5000, MA1-072, Thermo Fisher), and GAPDH (1:4000; sc-47724, Santa Cruz). Secondary antibodies: anti-mouse (1:4000; Dako), anti-rabbit (1:4000; Dako), NKX2-5 (1:1000, SC8697, Santa Cruz Biotech).

### Immunofluorescent Microscopy and associated antibodies

Mouse embryos or embryonic hearts were harvested at different stages and fixed in ice-cold PBS containing 4% paraformaldehyde overnight at 4°C. Fixed embryos were washed with PBS, then saturated in a 5, 10, 15, and 20% sucrose series in PBS, embedded in OCT Tissue-Tek mixed with 20% sucrose (1:1), and cut into 8-μm sections using a Leica CM 3050S cryostat (Leica Microsystems). Sections were permeabilized with wash buffer (PBS with 0.2% Triton 100), then blocked with blocking buffer (PBS with 1% BSA, 5% donkey serum, 0.2%Triton 100) for 1h at room temperature, followed by overnight incubation at 4°C with primary antibody diluted in blocking buffer. Sections were rinsed in wash buffer and incubated with fluorescently conjugated secondary antibodies and DAPI diluted in blocking buffer at room temperature for 1 hour, before rinsing and mounting in mounting buffer (Dako). Sections were imaged using an Olympus Fluoview FV1000 confocal microscope. Primary antibodies included: OGT (1:200, 61355, Active Motif), RL2 (1:300, MA1-072, Thermo Fisher), CD31 (1:150, 550274, BD Biosciences), NKX2-5 (1:50, SC8697, Santa Cruz Biotech), smooth muscle myosin heavy chain (1:200, BT-562, Alfa Aesar) and alpha actinin (1:400; 650931, Sigma), HIF1α (NB100-479, 1:200), TroponinT (Thermo Scientific, clone 13–11, 1:100).

### EdU incorporation assay and TUNEL staining

For EdU incorporation assay, pregnant female mice were given IP injection of EdU(Invitrogen) two hours prior to embryo dissection. After cryo-sectioning of embryo heart, EdU positive cells were detected according to manufacture’s introduction, which was followed by normal primary and secondary antibody incubation. Apoptotic cells in E12.5 heart sections were detected using TUNEL assay kit (Roche Cat. No. 11 684 795 910) according the manufacturer's protocol, witch was followed by immunostaining against alpha actinin to detect cardiomyocytes. Three embryos of each genotype were analyzed and at least five fields were analyzed for each section of the embryonic heart.

### Quantitative Real-Time PCR

Total RNA was isolated from homogenized embryonic hearts using TRIzol (Invitrogen) and reverse transcribed to cDNA using a Super Script III cDNA Synthesis Kit (Invitrogen), according to the manufacturer’s instructions. Complementary DNA amplicons were quantified by incorporation of SYBR Green probe (Biorad) into dsDNA. RT-PCR reactions were performed using Sso-Fast EvaGreen Real Time PCR (Bio-Rad) master mix in 96-well low profile PCR plates in the CFX96 Biorad Thermocycler. Samples were compared using the relative (comparative) Ct method after adjusting for *18s* RNA (ΔCt). Primers used in this study are listed in S2 Table.

### RNA Sequencing

Embryonic hearts at E11.5 were homogenized in TRIzol (Invitrogen) and total RNA was isolated according to the manufacturer’s instructions. cDNA libraries were prepared using an Illumina TruSeq stranded mRNA kit and subjected to deep sequencing. Reads were quality controlled with FastQC and mapped to the mm10 mouse reference genome with TopHat2 and Bowtie2. Differential expression of novel and reference transcripts was performed using Cuffdiff2. Resulting RNA-seq datasets can be accessed at GEO using accession number GSE146962.

### In Situ Hybridization

Whole-mount in situ hybridization was performed as previously described [15]. Briefly, embryos were fixed in pre-chilled 4% paraformaldehyde in phosphate-buffered saline at 4°C overnight, and subsequently subjected to RNA in situ analysis. The *Angiopoietin-1* in situ probe was generated from embryonic heart cDNA using the following primers: forward: TAGAACTAGTGGATCCCTGCCATTCTGACTCACAT, reverse: CGGTATCGATAAGCTTGTCTGTTGGAGAAGTTGCT.

### Pecam Whole Mount Staining

Whole mouse embryos or embryonic hearts were fixed in pre-chilled 4% paraformaldehyde in phosphate-buffered saline (PBS) at 4°C overnight. After washing with PBS, samples were dehydrated through serial methanol (form 25% to 100%), and then incubated in Dent’s bleach (methanol: dimethyl sulfoxide: 30%H2O2, 4:1:1) for 3 hours, followed by rehydration in serial methanol. After blocked in 3% skimmed milk, samples were then incubated with CD31 antibody (550274, BD Biosciences, dilution from 1:500 to 1:2000 depending on sample), and then followed by secondary antibody incubation. Then secondary antibody was detected by ABC kit (Vector Lab) and visualized using DAB substrates (Vector Lab) according to manufacture’s introduction.

### Ink injection

As described previously [20], Hearts were collected from mouse embryos at appropriate stages and dipped into heparinized PBS. Then, ink (Kiwa-Guro, Sailor, Japan) was gently injected from the ascending aorta using a glass micropipette. Samples were fixed in 4% paraformaldehyde solution and dehydrated in sequential ethanol solution. To clear samples, dehydrated samples were immersed in BABB (1:2 benzyl alcohol to benzyl benzoate).

### Statistical analysis

Data are presented as mean ± standard error of the mean (SEM). Statistical analysis was performed using Prism 6.0 software (GraphPad Software, La Jolla, CA), with two-tailed Student’s *t* test used for comparisons among groups as indicated. *P* values less than 0.05 were considered significant.

## Results

### Expression of OGT in embryonic and adult murine hearts

**Fig 1:**
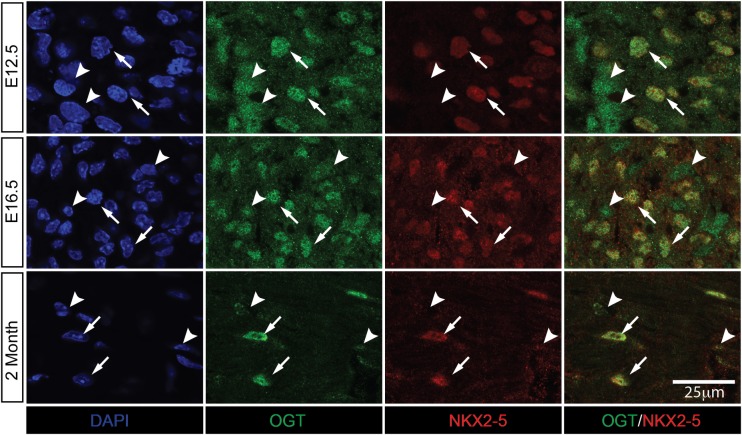
Nuclear localization of OGT in cardiomyocytes at different stages. Immunostaining of heart sections of E12.5, E16.5 embryos, and two months old mouse, with antibodies against OGT (green), NKX2-5 (red), and DAPI (blue).

To investigate the expression pattern of OGT in heart at different stages, we performed immunostaining for transverse heart sections at different stages. We used an antibody against NKX2-5, a cardiomyocyte specific transcription factor, to distinguish cardiomyocytes and non-cardiomyocytes. As shown in Fig 1, images were taken from left ventricle compact zone for E12.5 and E16.5 hearts, and from left ventricle free wall myocardium for adult heart (Fig 1). At E12.5, OGT was mostly restricted to the nucleus in cardiomyocytes, while in non-cardiomyocytes OGT had a relatively higher expression in cytosol (Fig 1). At E16.5, OGT was still mostly restricted to the nucleus in cardimyocytes. However, non-cardiomyocytes started to express less OGT compared to cardiomyocytes (Fig 1). At 2 months of age (adult), OGT was almost exclusively localized to the nucleus of cardiomyocytes. In contrast, OGT appeared to be greatly downregulated in non-cardiomyocytes (Fig 1). Our data suggested that OGT is expressed in nucleus of cardiomyocytes through heart development, indicating its function in heart development.

### Cardiomyocyte specific deletion of OGT leads to neonatal lethality

**Fig 2:**
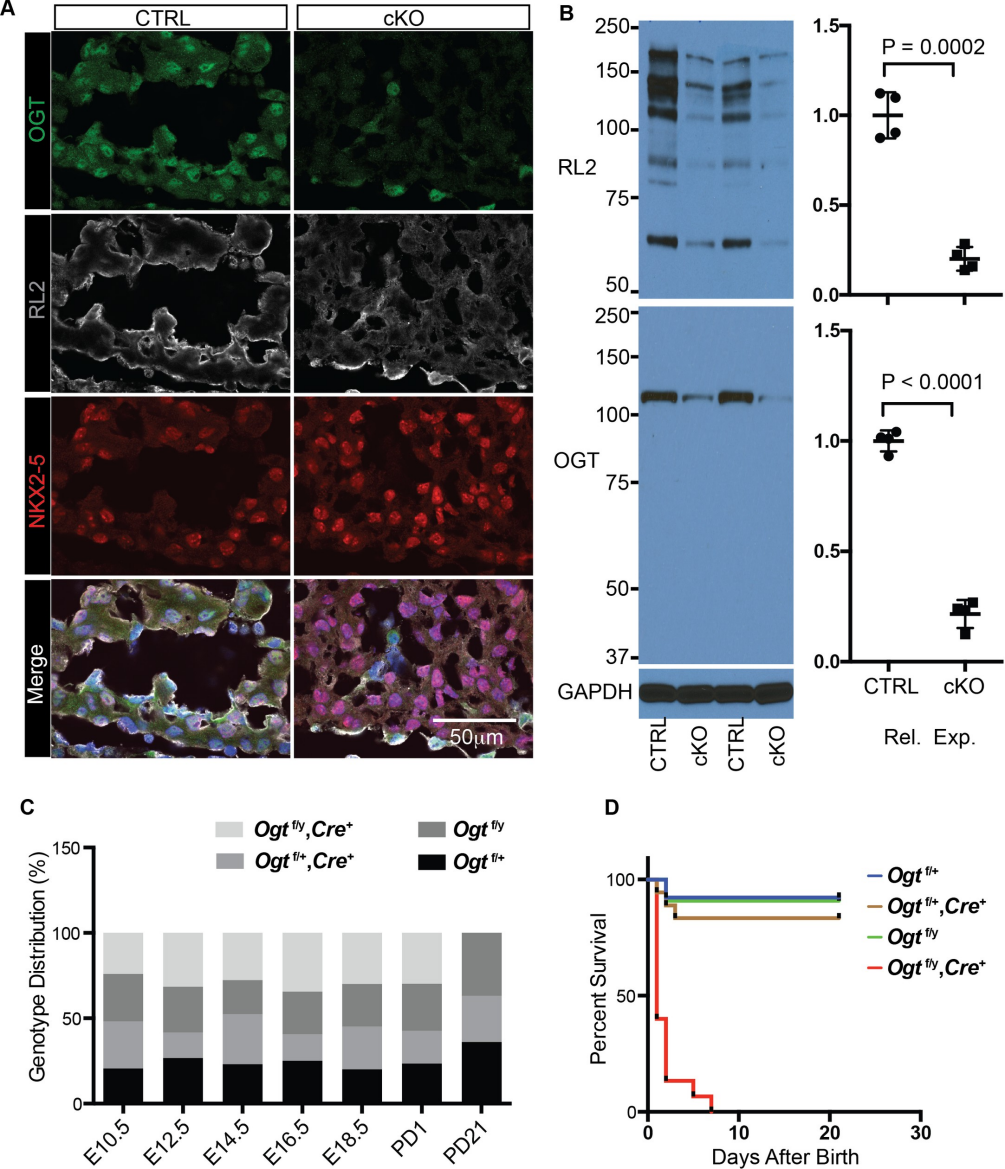
Efficient deletion of OGT in cardiomyocytes leads to postnatal lethality. **A**, Immunomicroscopic analysis of transverse sections of the heart of ctrl and cKO embryos at embryonic day E10.5, staining for OGT (green), O-GlcNAc (RL2, gray), NKX2-5 (red), and DAPI (blue). **B**, Western blot analysis of O-GlcNAc (RL2) and OGT of whole heart lysate from ctrl and cKO embryos at E14.5. N = 4 for each group. C, Genotype distribution (%) of embryos from *Ogt* ^f/f^ female and *TnT-Cre* male intercrosses at different stages. **D**, Postnatal surviving curve of mice with different genotypes.

A previous report showed that deletion of OGT using an alpha myosin heavy chain (*αMhc*) driven Cre affected postnatal viability [10], but there was variability in phenotypic severity. The variability could have arisen from the relatively late expression and low efficiency of *αMhc-Cre* as described in a recent review paper [21, 22]. To better explore the role of OGT during heart development, we crossed a *Troponin-T Cre* [17] (*TnT-Cre*) mouse line, which expresses Cre activity as early as E7.5 and gives high efficiency [17, 19, 22], with an *Ogt* floxed line to establish an early cardiomyocyte-specific OGT knockout mouse model (hereafter cKO). To confirm the efficiency of Cre-mediated ablation of *Ogt*, we performed both immunostaining and western blot analyses using a polyclonal antibody against OGT, and a monoclonal antibody against global O-GlcNAc modification (RL2). From immunostaining results, almost all NKX2-5 postive cardiomyocytes showed no signal for OGT in cKO hearts, while cardiomyocytes in control hearts showed strong nuclear staining for OGT. In contrast, NKX2-5 negative non-cardiomyocytes in both control and knockout hearts had similar levels of OGT signal. Similarly, RL2 staining was diminished in knockout myocardium compared to that in control myocardium (Fig 2A,S1 Fig). Western blot analyses using whole heart samples showed that levels of OGT protein and global O-GlcNAc were reduced by approximately 80% in cKO hearts when compared to control hearts (Fig 2B).

Similar to, but more severe than previously reported [10], we did not recover any knockout mouse at weaning stages (Fig 2C and S1 Table). Genotypic analyses revealed that at E18.5 knockout embryos were still viable and recovered at expected Mendelian ratios. At postnatal day 1, although knockout mice were still observed at Mendelian ratios, nearly half of them (6 of 14) had died (Fig 2C and S1 Table). Further survival analysis showed that a majority of cKO mice (11 out 13) died two days after birth, and no knockout mice survived beyond 7 days after birth. These data suggested that ablation of OGT in cardiomyocytes led to postnatal lethality shortly after birth.

### Loss of OGT leads to dilated cardiomyopathy

**Fig 3:**
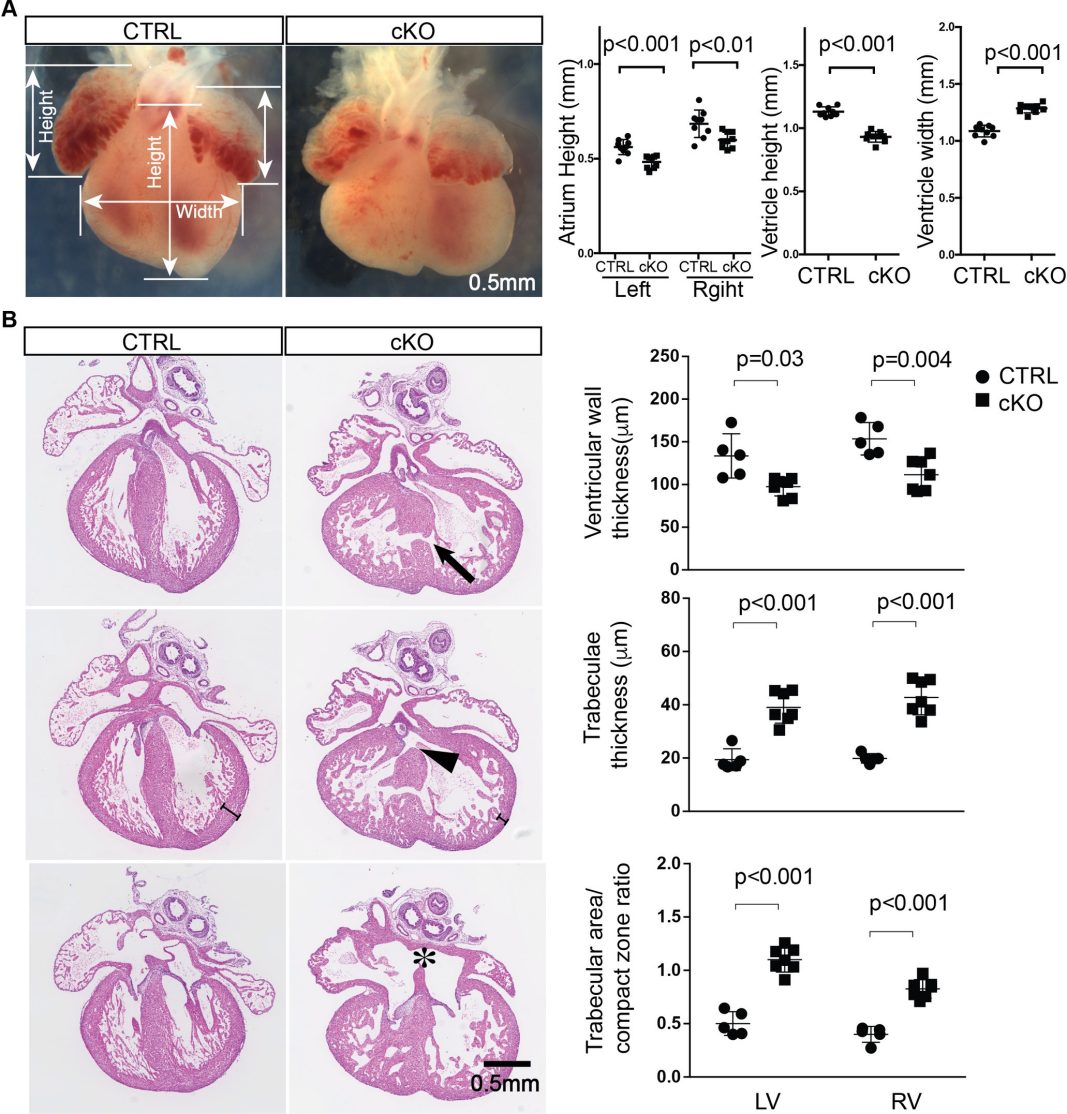
Loss of OGT in cardiomyocytes results in dilated cardiomyopathy with multiple heart developmental defects. **A**, Microscopic assessment of ctrl and cKO hearts at E16.5 (left panel), and quantitative analysis of atrial height, ventricular height and width (right panel). N = 5 for control group and N = 7 for cKO group. **B**, Histological evaluation with hematoxylin and eosin staining of ctrl and cKO hearts at E16.5 (left panel), arrowhead, arrow and asterisk indicates membranous ventricular septal defect (VSD), muscular VSD, and atrial septal defect (ASD) respectively. Quantitative comparison of ctrl and cKO heart ventricular wall thickness, trabecular thickness, and trabeculae/compact zone ratio are shown in right panel. N = 5 for control group and N = 7 for cKO group.

When compared to control hearts at E16.5, cKO hearts showed dramatic morphological differences (Fig 3A). Both left and right atria of cKO hearts were smaller than those of control hearts, and showed an uneven surface. The height of knockout heart ventricles was smaller than that of control heart ventricles, whereas the width of knockout heart ventricles was larger than that of control heart ventricles. Histological analysis revealed that loss of OGT in cardiomyocytes caused multiple cardiac developmental defects, including dilated ventricles, atrial septal defects, membranous and muscular ventricular septal defects, reduced ventricular wall thickness, and increased trabecular area and thickness (Fig 3B). Careful quantification analysis confirmed reduced wall thickness, and increased trabecular area (Fig 3B). At the whole mount level, overall morphological differences were apparent by E12.5 (S2 Fig), with histological analyses revealing differences in wall thickness and trabecular thickness as early as E11.5 (S3 Fig). Collectively, those data suggested that OGT was essential for heart development, with loss of OGT in early cardiomyocytes leading to dilated cardiomyopathy with multiple cardiac defects.

### Loss of OGT causes developmental defects in coronary vessel formation

**Fig 4:**
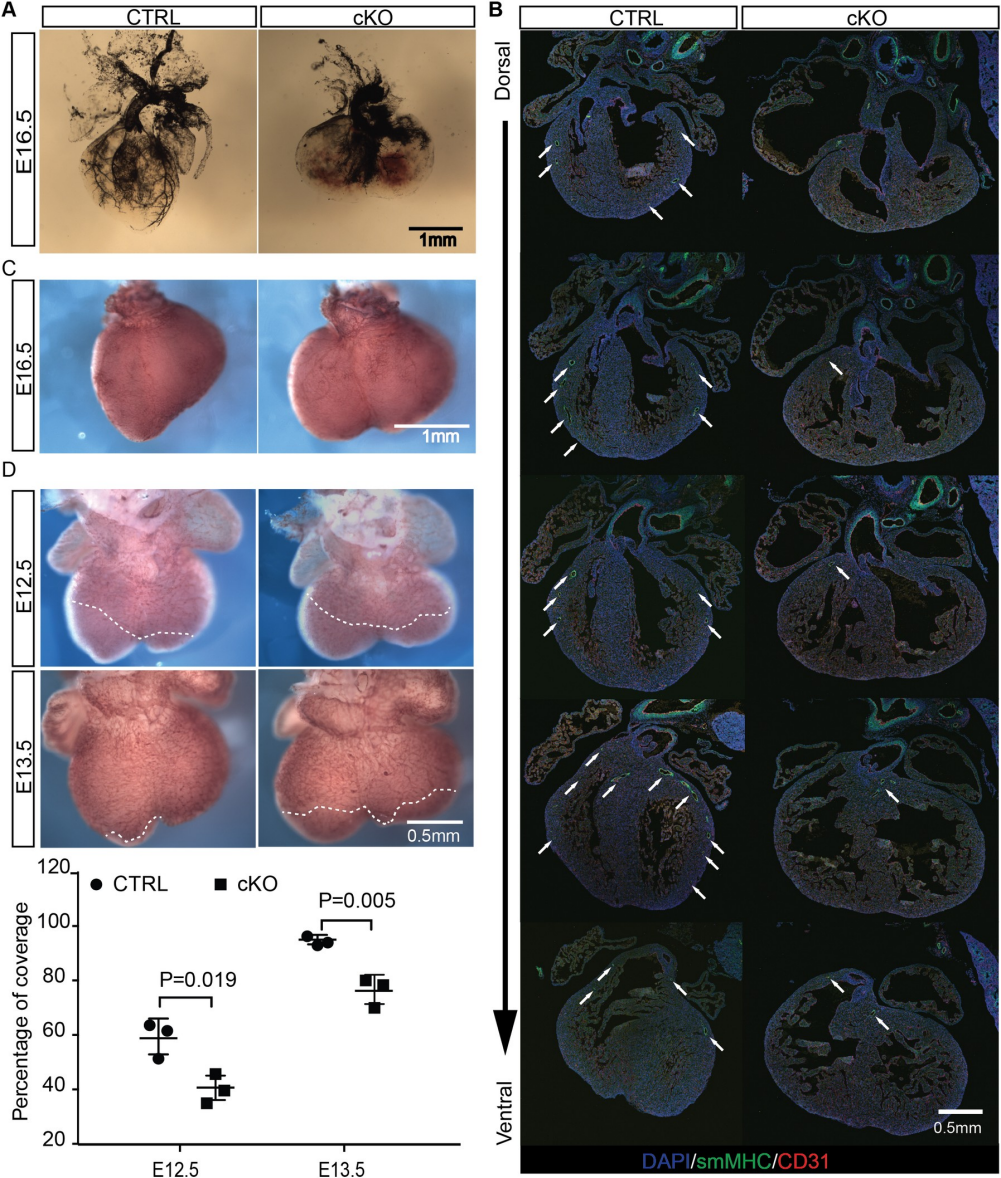
Loss of OGT alters coronary vessel development. **A**, Visualization of coronary arteries in ctrl and cKO hearts by ink injection at E16.5. **B**, Immunostaining of consecutive sections of ctrl and cKO hearts at E16.5 using antibodies against sm-MHC (green), CD31 (red), and DAPI (blue). **C**, Dorsal view of whole mount PECAM staining of E16.5 ctrl and cKO hearts. **D**, Dorsal view of whole mount PECAM staining of E12.5 and E13.5 ctrl and cKO hearts. The front line of coronary plexus is indicated by the dashed line (upper panel). The quantitative analysis of percentage of plexus coverage is shown in lower panel. N = 3 for each group.

From morphological and histological analyses, coronary arteries were barely evident in cKO hearts at E16.5 (Fig 3). To confirm this, angiography based on ink injections from the aorta were performed. This analysis further demonstrated that coronary artery formation was severely affected in cKO hearts (Fig 4A). In control hearts, coronary arteries were well formed and extended from the aorta to the apex, while in cKO hearts, the expansion of the coronary arteries was largely blocked. Larger vessels, which were clearly evident in control hearts, were largely absent in cKO hearts. Instead, there were many more small vessel branches formed in cKO hearts. Furthermore, immunostaining of serial heart sections using antibody against smooth muscle myosin heavy chain (smMHC), a marker of smooth muscle cells in large arterial vessels, confirmed that coronary artery formation was dramatically affected in cKO hearts. In control hearts, smMHC positive cells were detected throughout the ventricular walls. In contrast, in cKO hearts, smMHC positive cells were only detected at the area close to the heart base (Fig 4B). Although whole mount PECAM staining at E16.5 did not show differences in coronary vein development between control and cKO hearts (Fig 3C), whole mount PECAM staining of E12.5 and E13.5 hearts demonstrated that early coronary plexus formation was delayed by loss of OGT (Fig 4D). Our data suggested that loss of OGT in cardiomyocytes alters the development of coronary vessel, specially the development of coronary artery.

### Cardiomyocyte proliferation, but not apoptosis, is affected by loss of OGT

**Fig 5:**
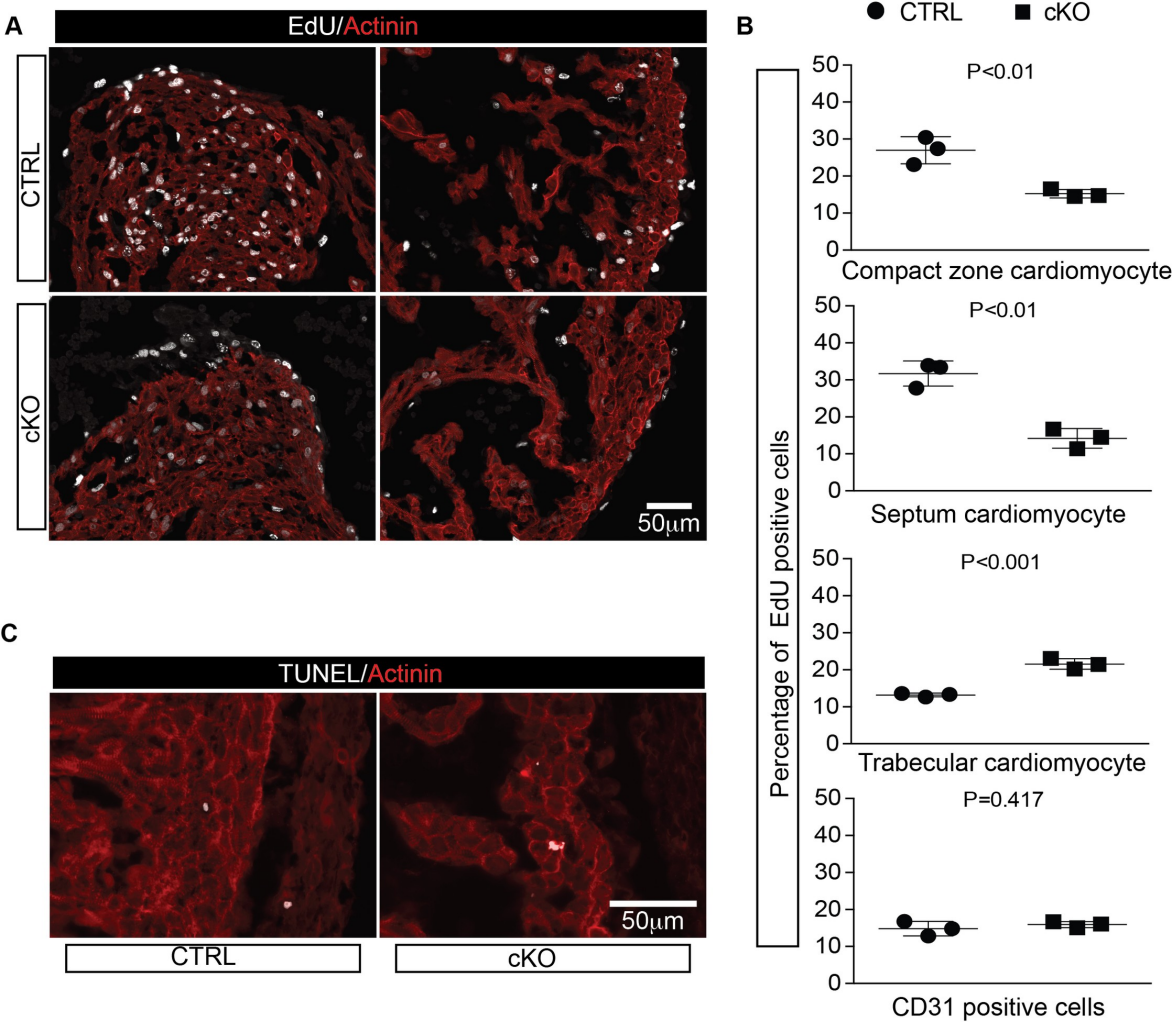
Loss of OGT alters cardiomyocytes proliferation. **A**, EdU assay analysis of ctrl and cKO hearts at E12.5. Staining for EdU (gray) and alpha-actinin (red) are shown. **B**, Quantitative analysis of EdU positive rate for compact zone cardiomyocytes, septum cardiomyocytes, trabecular cardiomyocytes, and CD31 positive endothelium cells. N = 3 for each group. **C**, Representative images of TUNEL staining of transverse sections of ctrl and cKO hearts at E12.5. Staining for TUNEL (gray) and alpha-actinin(red) are shown.

As ventricular wall thickness and trabecular thickness were affected when OGT was deleted from early cardiomyocytes, we performed an EdU incorporation assay at E12.5 to investigate whether cardiomyocyte proliferation was affected or not. Consistent with observed phenotypes, proliferation assays showed that cardiomyoctye proliferation in both compact zone and ventricular septum was significantly reduced. In compact zone and ventricular septum of control hearts, the percentage of cardiomyocytes with EdU incorporation following two hours of labeling were 26.98 ± 2.118 and 31.69 ± 1.964, which was reduced to 15.25 ± 0.65 and 14.20 ± 0.511 respectively in cKO hearts (Fig 5A and 5B). However, the EdU incorporation rate was increased in trabeculae of cKO hearts (21.58±0.836%) compared to trabeculae of control hearts (13.21 ± 0.2978%) (Fig 5A and 5B). Furthermore, TUNEL staining did not show significant difference between control and cKO hearts (Fig 5C,S4 Fig). Taken together, those data indicated the decreased cardiomyocyte proliferation rather than abnormal apoptosis induced by loss of OGT contributed to those phenotype observed above.

### Loss of OGT in cardiomyocytes causes transcriptome changes

Since O-GlcNAc has been linked to transcriptional regulation, we did whole transcriptome analysis using RNA sequencing. We chose E11.5 cKO and littermate control hearts for RNA-seq, as E11.5 was the time point when phenotypes were first evident in knockout hearts. RNA-seq revealed that there were a total of 753 dysregulated genes (fold-change greater than 1.5). Among those genes, 413 genes were upregulated, and 340 genes were downregulated (S5A Fig). We also used the bioinformatics database DAVID to do gene ontology analysis. Biological process analysis showed that most genes upregulated consequent to loss of OGT were related to morphogenesis, homeostasis, and muscle contraction (S5B Fig). These results were consistent with cardiac morphological phenotypes observed. Unexpectedly, there were also some categories related to vesicle membrane that were also upregulated. Similar analysis was also performed for downregulated genes. Among downregulated biological process categories, the most highly significant was metabolic processes (S5C Fig). Using quantitative real-time PCR (qRT-PCR), we validated a subset of genes, which were shown to be dysregulated by RNA-seq (S5D Fig). The qRT-PCR results were consistent with RNA-seq analysis. Our RNA-seq analysis suggested that loss of OGT resulted in global alterations in gene transcription.

### Loss of OGT decreases angiopoietin-1 expression in cardiomyocytes

**Fig 6:**
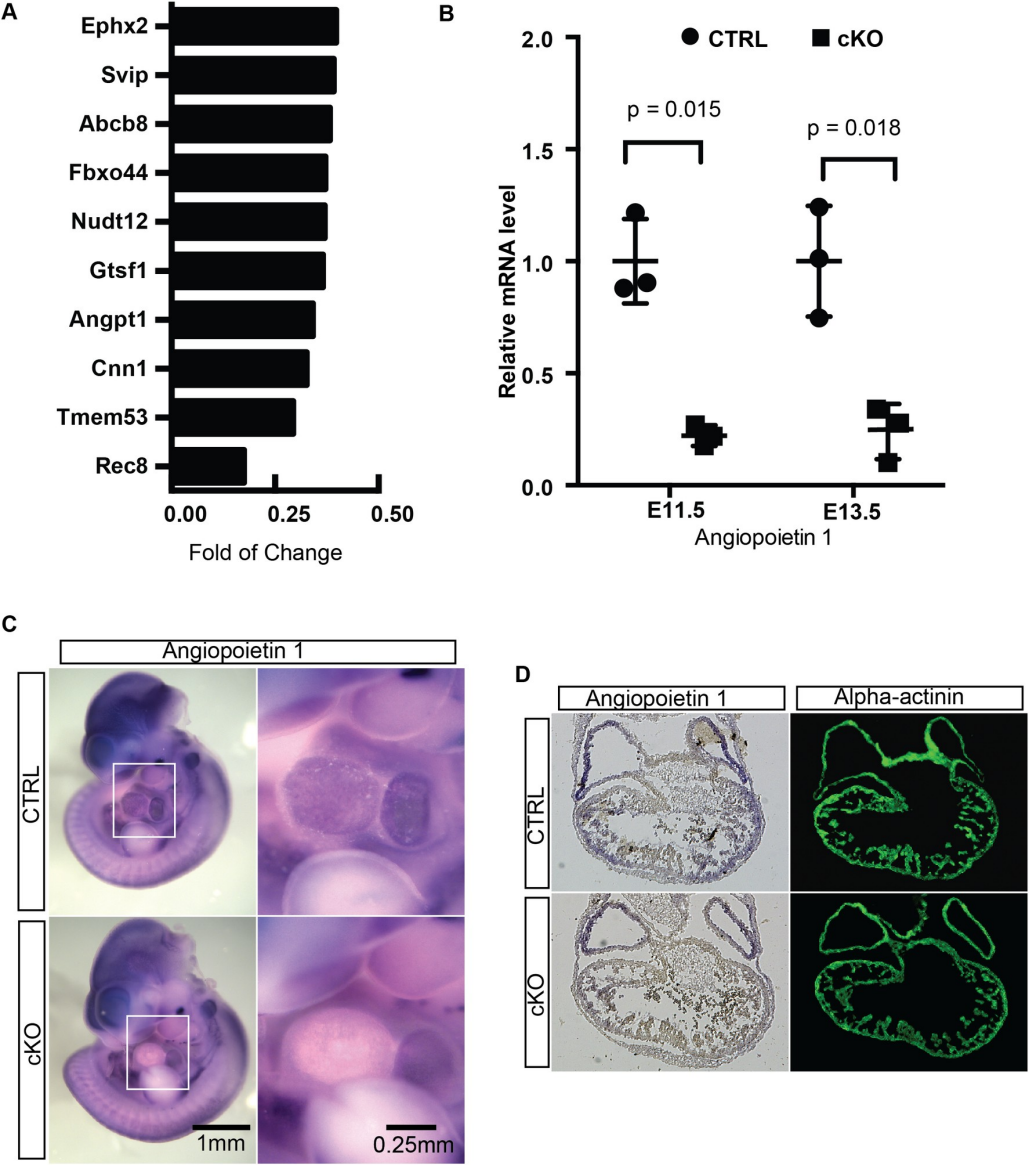
Loss of OGT downregulates Angiopoietin1 expression. **A**, The top 10 down-regulated genes revealed by RNA sequencing including Angpt1. **B**, Real time PCR confirmation of Angpt1 downregulation at stages of E11.5 and E13.5. **C**, Whole mount *in situ* hybridization for Angpt1 in ctrl and cKO embryos at E10.5 (left panel). And high magnification views of boxed cardiac area are shown in right panel. **D**, Immunostaining analysis of transverse sections of embryos described in Fig 6C with antibody against alpha-actinin.

Examination of the top downregulated genes with significant levels of expression (mean RPKM in control hearts greater than 2) revealed that angiopoietin-1 (Angpt1), an essential gene for heart development [13, 15, 16], was dramatically decreased (Fig 6A). Using qRT-PCR we confirmed that Angpt1 was downregulated approximately 4-fold (Fig 6B). To further confirm downregulation of Angpt1, we performed RNA *in situ* hybridization at E10.5. Angpt1 was highly expressed in hearts of control embryo. In contrast, the signal for Angpt1 was specifically lost in hearts of cKOs (Fig 6C). Immunostaining of transverse sections with antibody to alpha actinin demonstrated that expression of Angpt1 was mainly restricted to cardiomyocytes, indicating that OGT regulated expression of Angpt1 in a cell-autonomous manner.

Since the oxygen-sensitive transcriptional activator hypoxia-inducible factor-1 (HIF-1) is a key transcriptional mediator of angiogenesis related genes including Angpt1 [23], and cardiomyocyte specific knockout of HIF-1α in mouse leads to embryonic lethality [24], we performed immunostaining to check the exrepssion level and localization of HIF-1α. Our data showed that loss of OGT in cardiomyocytes affected neither the expression nor localization of HIF-1α in cardiomyocytes (S6 Fig). NKX2-5, an essential transcription factor in cardiomyocytes, has been reported to regulate Angpt1 in fetal cardiomyocytes in a NKX2-5 hypomorphic mouse model [25]. And our RNA in situ hybridization also confirmed that Angpt1 was dramatically downregulated in NKX2-5 knockout embryos at E9.0 (S7A Fig). However, we were unable to detect the change of expression level of NKX2-5 in cKO cardiomyocytes (S7B Fig). Also, we could not detect any O-GlcNAcylation of NKX2-5 using immunoprecipitation method (S7C Fig). So our data indicates that OGT is not likely to regulate Angpt1 through HIF-1α or NKX2-5.

## Discussion

In our study, all *Tnt-Cre* mediated OGT knockout mice died before 1 week of age, which is similar to, but more severe than the phenotype observed using *αMhc-Cre* [10]. This may reflect the fact that *Tnt-Cre* expresses earlier and is more efficient than *αMhc-Cre* [22]. Our results suggest that *Tnt-Cre* might be a better choice to study requirements for OGT during early heart development. Our results demonstrated that loss of OGT in cardiomyocytes caused multiple cardiovascular abnormalities, including hypertrabeculation, biventricular dilation, atrial septal defects, ventricular septal defects, and defects in coronary vessel development. EdU incorporation assays showed that loss of OGT in early cardiomyocytes resulted in decreased proliferation of compact zone cardiomyocytes, while proliferation of trabecular cardiomyocytes was increased. These data were consistent with the observed cKO phenotype, with mutants exhibiting thinner compact zones and thicker trabeculae relative to controls. RNA-seq data showed that ablation of OGT altered expression of a large set of genes. A majority of upregulated genes were related to contractile function, which may reflect myocardial remodeling consequent to loss of OGT. In contrast, downregulated genes were related to regulation of metabolism, including mitochondrial function.

Angpt1 was significantly downregulated in OGT cKO hearts. Angpt1 is highly expressed in developing cardiomyocytes [13, 16], and loss of Angpt1 in cardiomyocytes leads to embryonic lethality around E14.5, with phenotypes very similar to those observed in our cKO hearts, including increased myocardial trabeculation, thinner compact zone, ventricular septal defects and abnormal coronary vessel development [15], albeit more severe, perhaps reflecting a complete loss of function of Angpt1 mutants, rather than the 75% reduction in Angpt1 mRNA levels observed in our cKOs. Different from previous reports that loss of Angpt1 in cardiomyocytes specifically affected subepicardial coronary veins, but not intramyocardial arteries [15], loss of OGT mainly affected coronary artery development. We speculate that the differences observed in the two mutants can be attributed to differences in the levels of Angpt1 that are present. For example, low levels of Angpt1 in myocardium in OGT cKOs may be sufficient for subepicardial coronary vein development. However, the low level of Angpt1 in cKOs may be insufficient for coronary artery remodeling. It is also possible that defects in coronary artery development do not reflect reductions in Angpt1, but are secondary to an overall developmental delay in the mutants, although given the known role of Angpt1 we feel this is less likely.

Although HIF-1α is considered one of the key transcription factors controlling angiogenesis through regulating expression of related genes including Angpt1, our data demonstrated that loss of OGT did not affect expression or localization of HIF1α, suggesting that OGT did not regulate Angpt1 through HIF1α. These data are consistent with a recent study, in which Angpt1 was not among genes dysregulated consequent to loss of cardiomyocyte HIF1α [24]. Our data (S7A Fig) together with a previous report [25] demonstrated that NKX2-5 is upstream of Angpt1 expression in fetal cardiomyocytes. NKX2-5 has been shown to be O-GlcNAcylated when overexpressed in HEK293 cells [26]. However, NKX2-5 expression levels were unchanged in cKO hearts, and we were unable to detect O-GlcNAcylation of NKX2-5 in embryonic heart. Thus, we could find no data to support the idea that OGT regulated Angpt1 through NKX2-5 in cardiomyocytes. Further investigation will be required to determine mechanisms by which Angpt1 is regulated by OGT in cardiomyocytes.

Of note, since *Ogt* is an X-linked gene, and we crossed Ogt^f/f^ females with male *Tnt-Cre* transgenic mice to obtain knockouts, our approach only generated male knockouts. To obtain female knockouts, we would have had to cross Ogt^f/y^ males with TnTCre^+^;Ogt^f/+^ females. However, we have noted that the *Tnt-Cre* can be leaky in the female germline. This, in combination with the expected low frequency of mutant females (12.5%), led to our focusing our studies on male knockouts. Future investigation would be required to determine whether the phenotype we observed in gender dependent.

In summary, consequent to loss of OGT in cardiomyocytes, mice developed multiple cardiac abnormalities, and subsequent early postnatal lethality. Our data indicated that OGT plays an essential role in regulating heart development through activating expression of cardiomyocyte Angpt1.

## Supporting information

S1 Fig Whole section image of immunomicroscopic analysis of Cre effieciency in cardiomyocytes. Transverse sections of the hearts in ctrl and cKO embryos at embryonic day E10.5, were stained for OGT (green), O-GlcNAc (RL2, gray), NKX2-5 (red) and DAPI (blue). High magnification images of boxed areas are shown in Fig 2A.
(TIF)

S2 Fig Morphologic assessment of control and cKO hearts at early development stages. (TIF)

S3 Fig Histological assessment of control and cKO hearts at early development stages. (TIF)

S4 Fig Quantitative analysis of TUNEL positive cardiomyocytes at E12.5 for both control and cKO embryos. The percentage of TUNEL positive cardiomyocytes was analyzed for compact zone, trabaculae, and septum separately. N = 3 for each group.
(TIF)

S5 Fig Comparison of transcriptomes for control and cKO hearts. **A**, Heatmap analysis of differentially expressed genes (> 1.5 fold change) in ctrl and cKO hearts at E11.5. **B** and **C**, Gene ontology analysis of upregulated (B) and downregulated (C) genes respectively. **D**, Verification of RNA-seq data for selected genes using qRT-PCR.
(TIF)

S6 Fig Immunostaining of HIF-1α for control and cKO hearts at E12.5. Cross sections of ctrl and cKO hearts at E12.5 were stained with DAPI and antibodies against HIF-1α (green), Troponin-T (red).
(TIF)

S7 Fig NKX2-5 regulates Angipoietin-1, and interacts with OGT but shows undetectable O-GLcNAcylation. **A**, In situ hybridization of Angiopoietin-1 for wild type and NKX2-5 null embryos at E9.0. Heart areas are indicated by dashed lines. **B**, Immunoprecipitation with NKX2-5 antibody using E14.5 control and cKO heart lysate followed by immunoblotting using antibodies against O-GlcNAc (RL2) and NKX2-5 respectively. H.C., heavy chain; L.C., light chain. **C**, Immunostaining of cross section of E12.5 control and cKO hearts. DAPI (blue), OGT (green) and NKX2-5 (red).
(TIF)

S1 Table Genotype frequencies at different stages. (DOCX)

S2 Table Primers used for qRT-PC. (DOCX)
