# Supplementary figures and images for: O-linked β-N-acetylglucosamine transferase plays an essential role in heart development through regulating angiopoietin-1
Source: PLoS Genet. 2020 Apr 6;16(4):e1008730. doi: 10.1371/journal.pgen.1008730 (PMC7182263; doi:10.1371/journal.pgen.1008730)

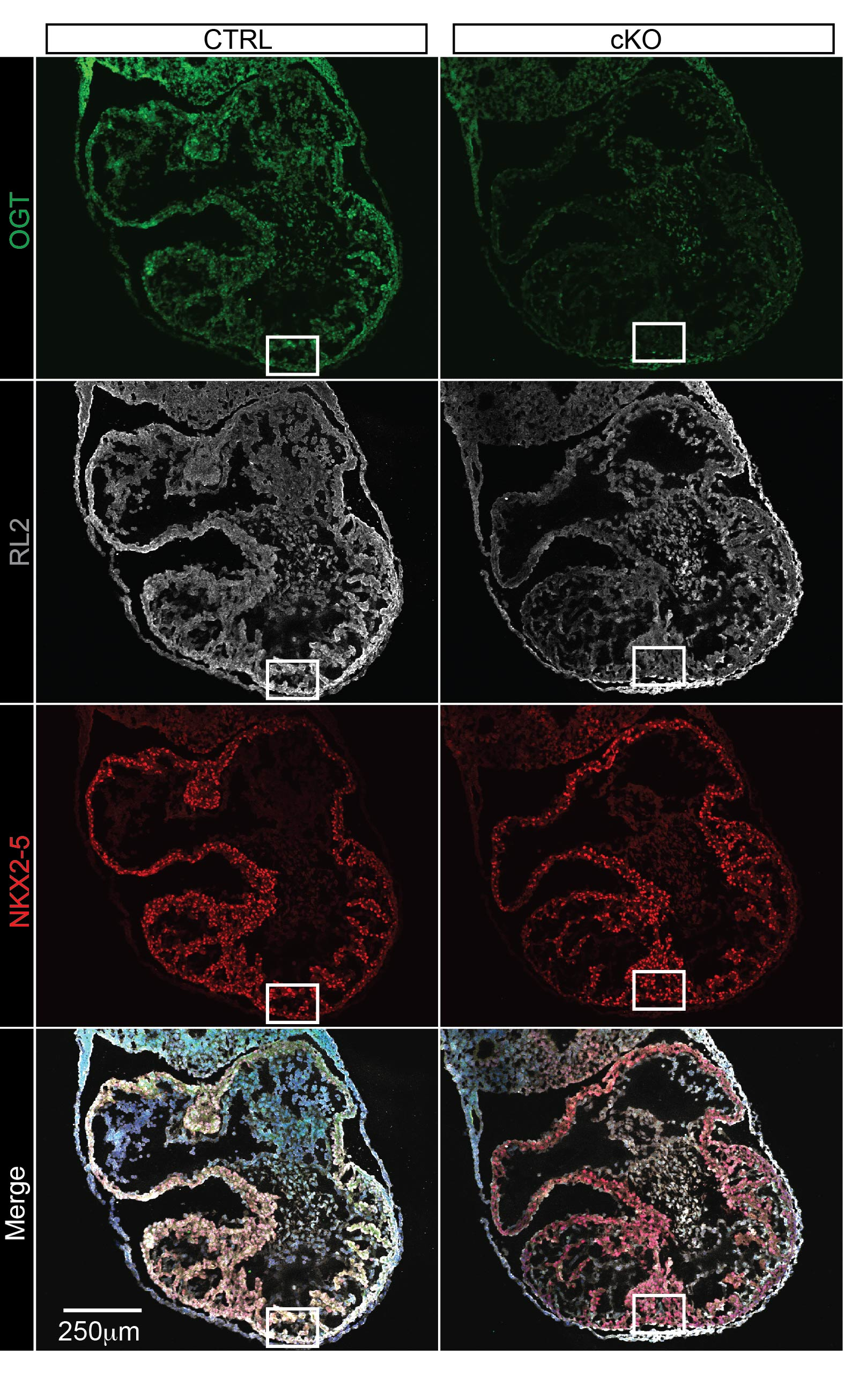

Supplement: S1 Fig — Transverse sections of the hearts in ctrl and cKO embryos at embryonic day E10.5, were stained for OGT (green), O-GlcNAc (RL2, gray), NKX2-5 (red) and DAPI (blue). High magnification images of boxed areas are shown in Fig 2A. (TIF) [file pgen.1008730.s001.tif]

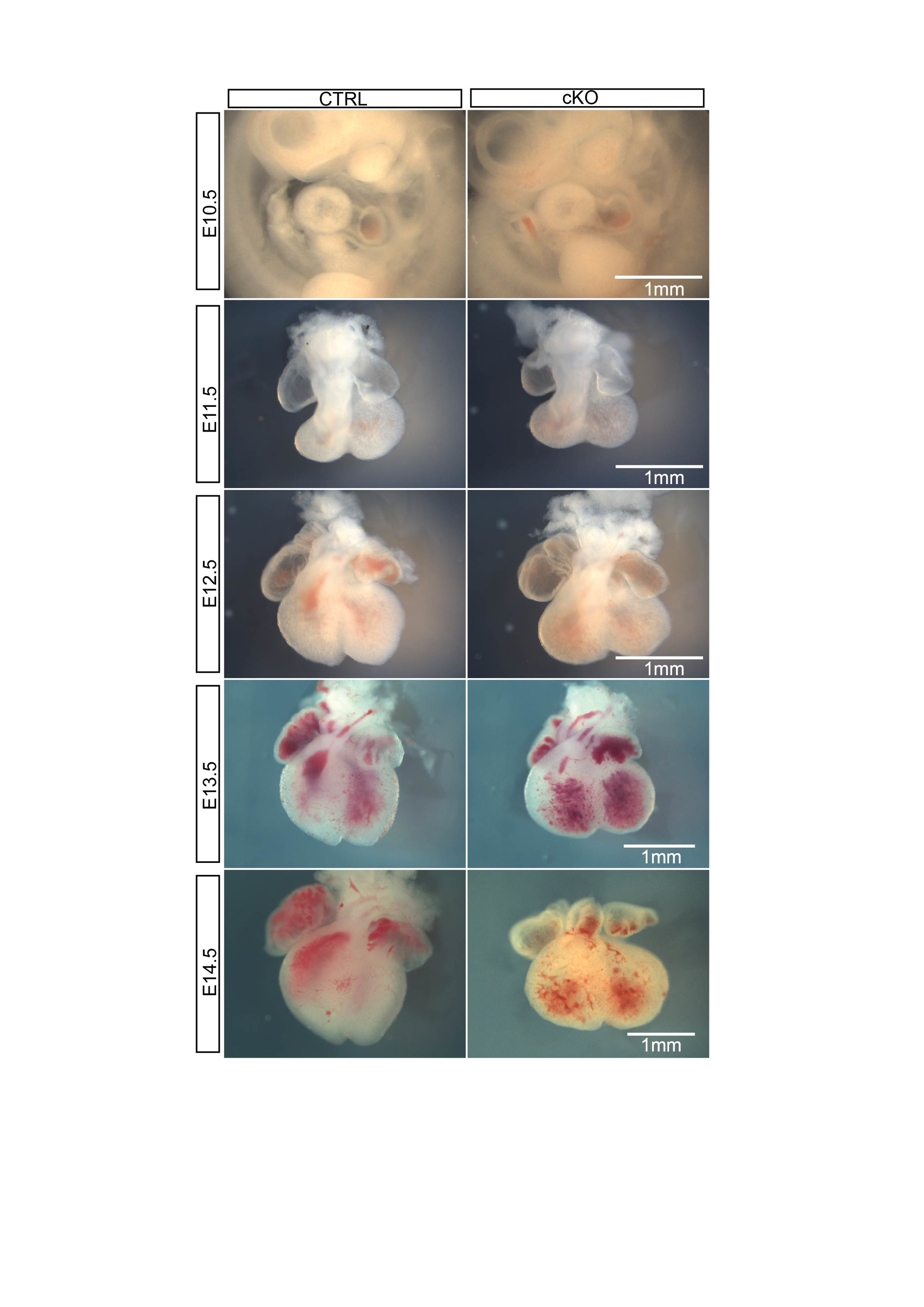

Supplement: S2 Fig — (TIF) [file pgen.1008730.s002.tif]

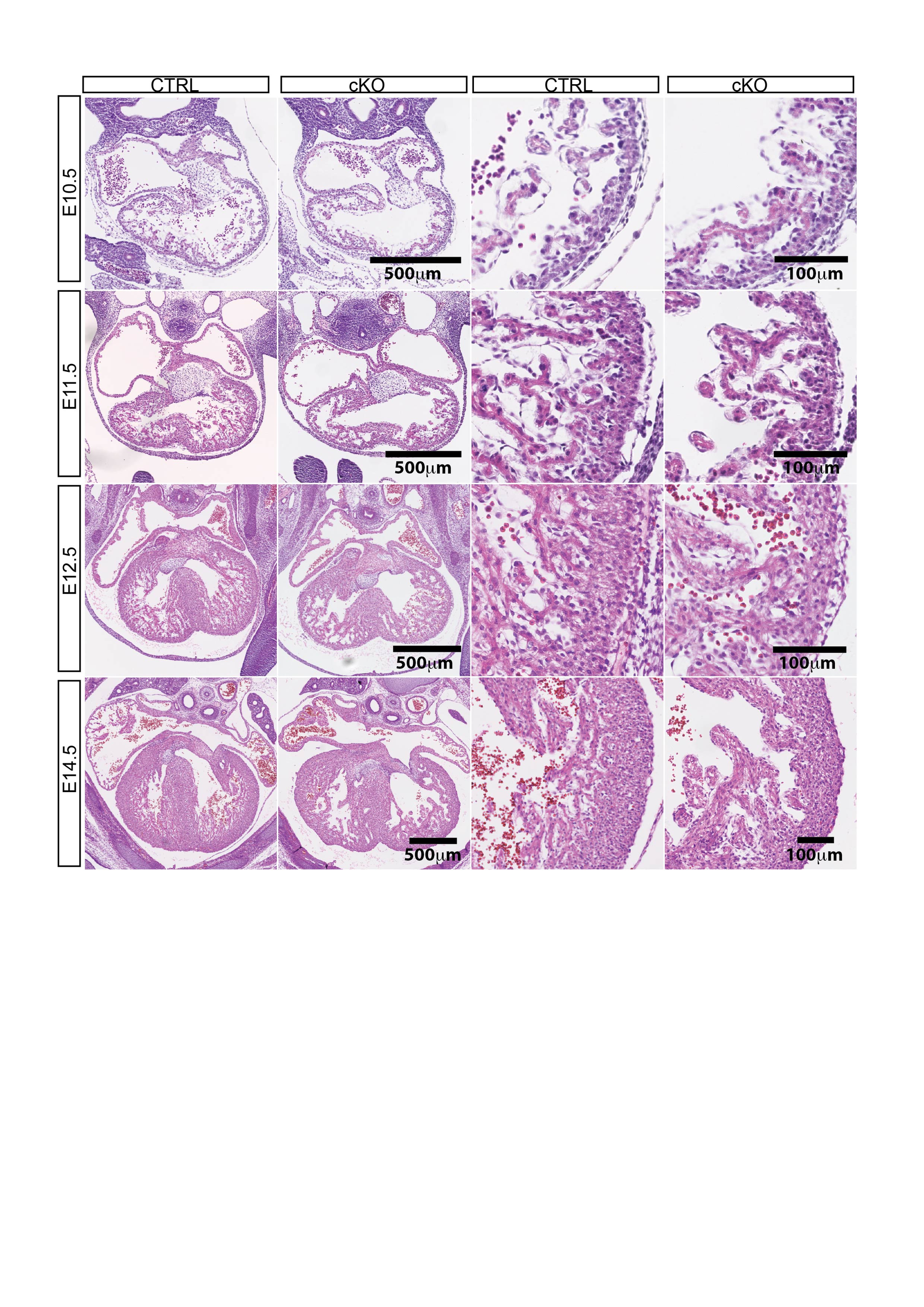

Supplement: S3 Fig — (TIF) [file pgen.1008730.s003.tif]

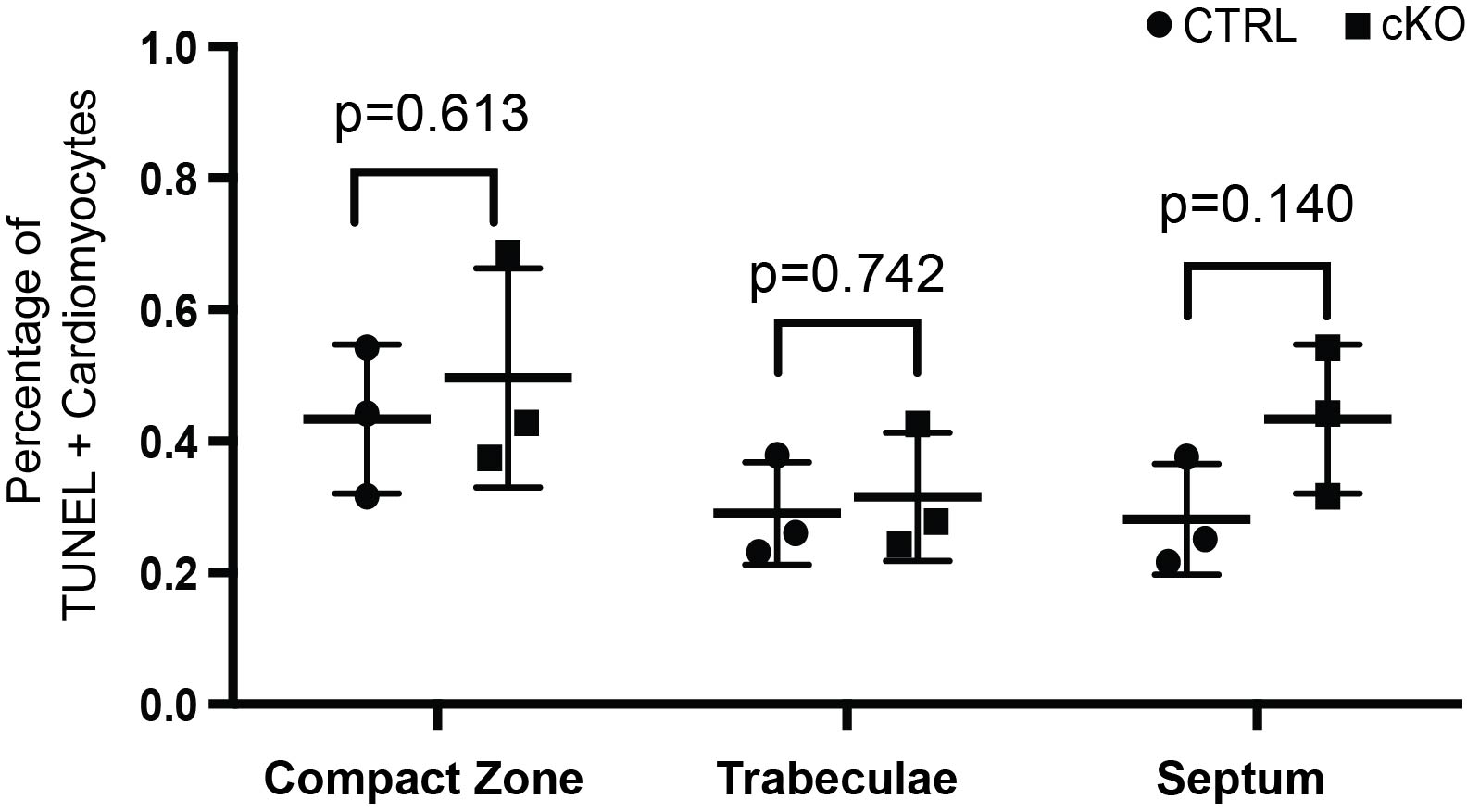

Supplement: S4 Fig — The percentage of TUNEL positive cardiomyocytes was analyzed for compact zone, trabaculae, and septum separately. N = 3 for each group. (TIF) [file pgen.1008730.s004.tif]

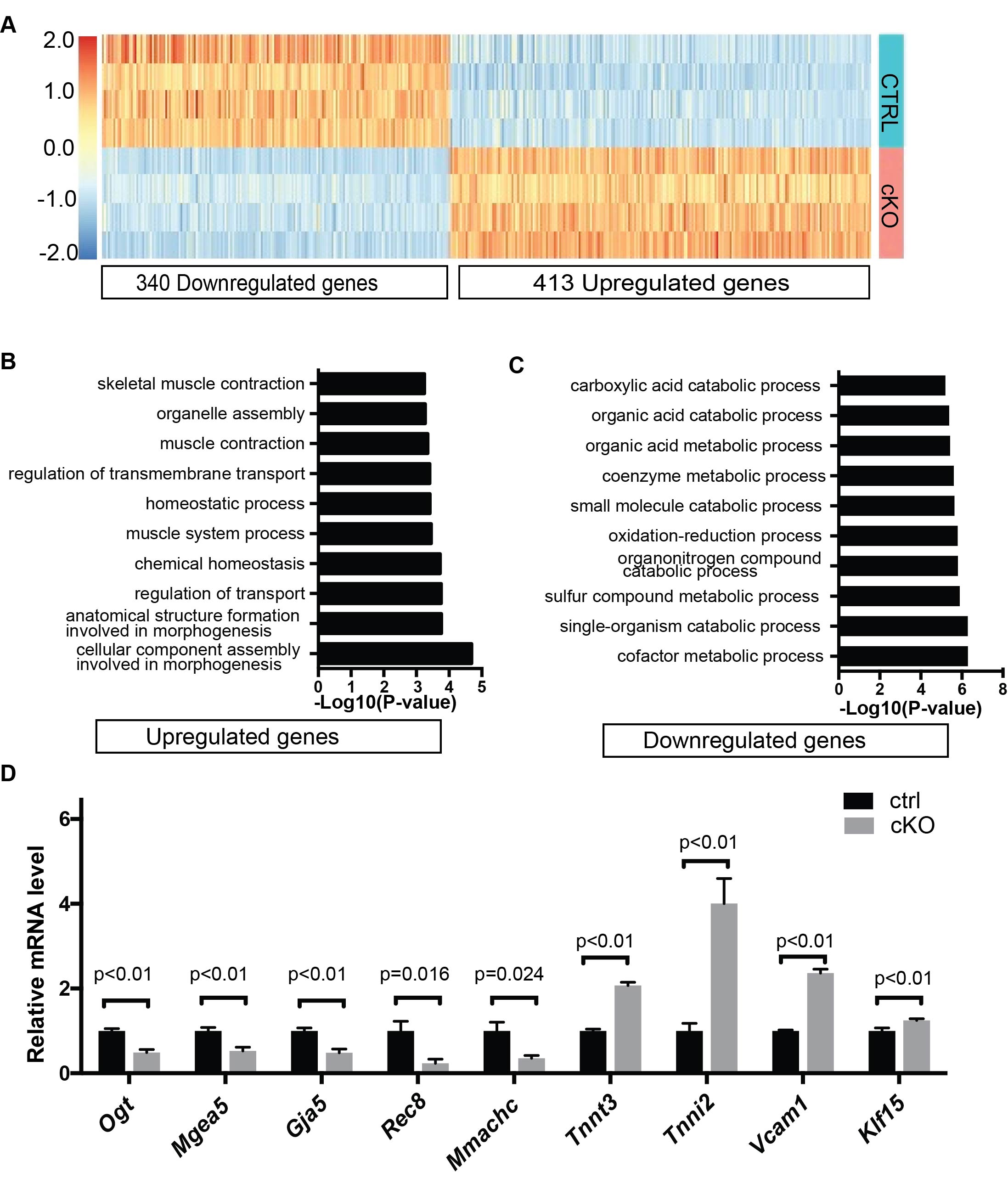

Supplement: S5 Fig — A, Heatmap analysis of differentially expressed genes (> 1.5 fold change) in ctrl and cKO hearts at E11.5. B and C, Gene ontology analysis of upregulated (B) and downregulated (C) genes respectively. D, Verification of RNA-seq data for selected genes using qRT-PCR. (TIF) [file pgen.1008730.s005.tif]

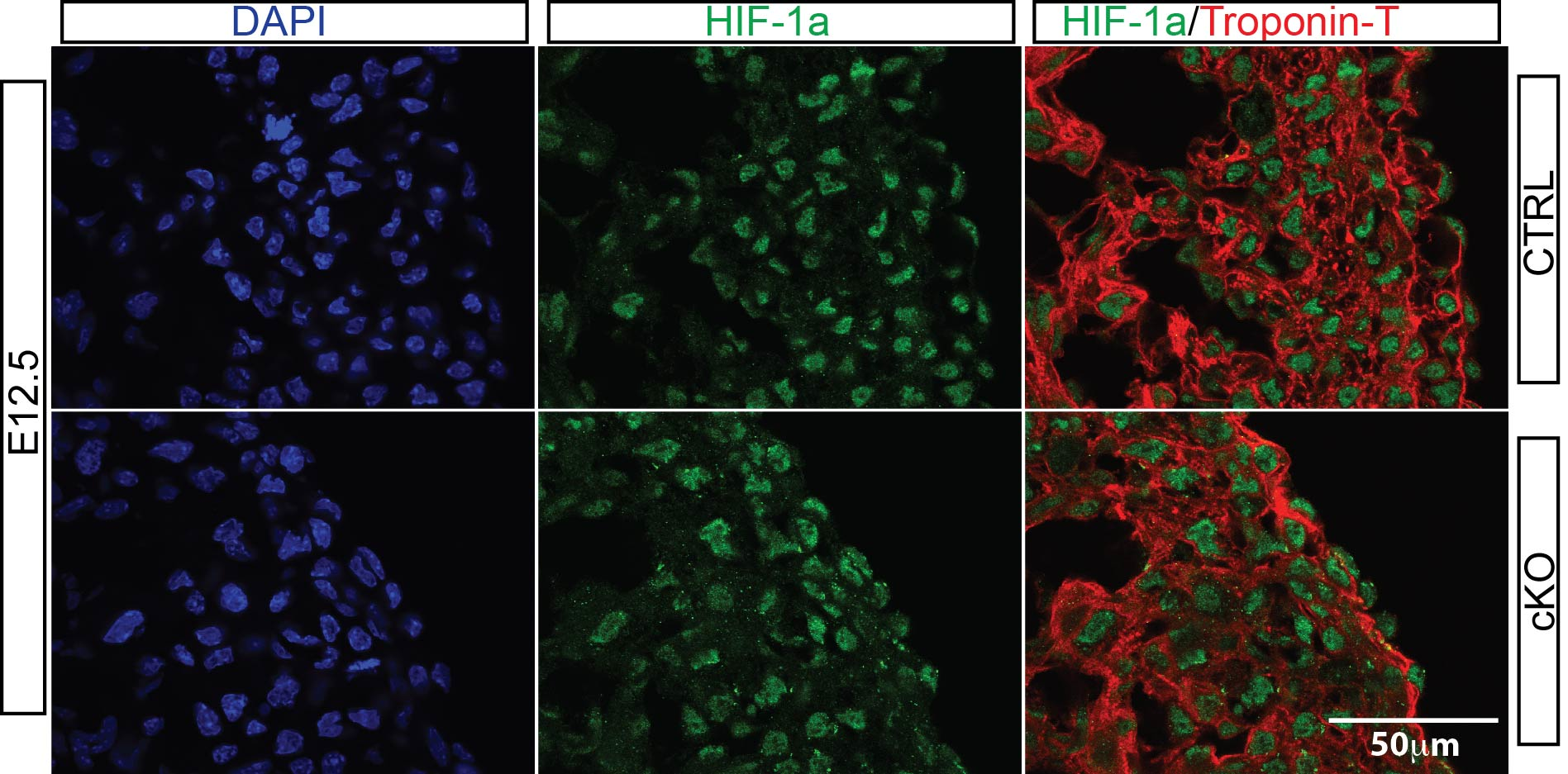

Supplement: S6 Fig — Cross sections of ctrl and cKO hearts at E12.5 were stained with DAPI and antibodies against HIF-1α (green), Troponin-T (red). (TIF) [file pgen.1008730.s006.tif]

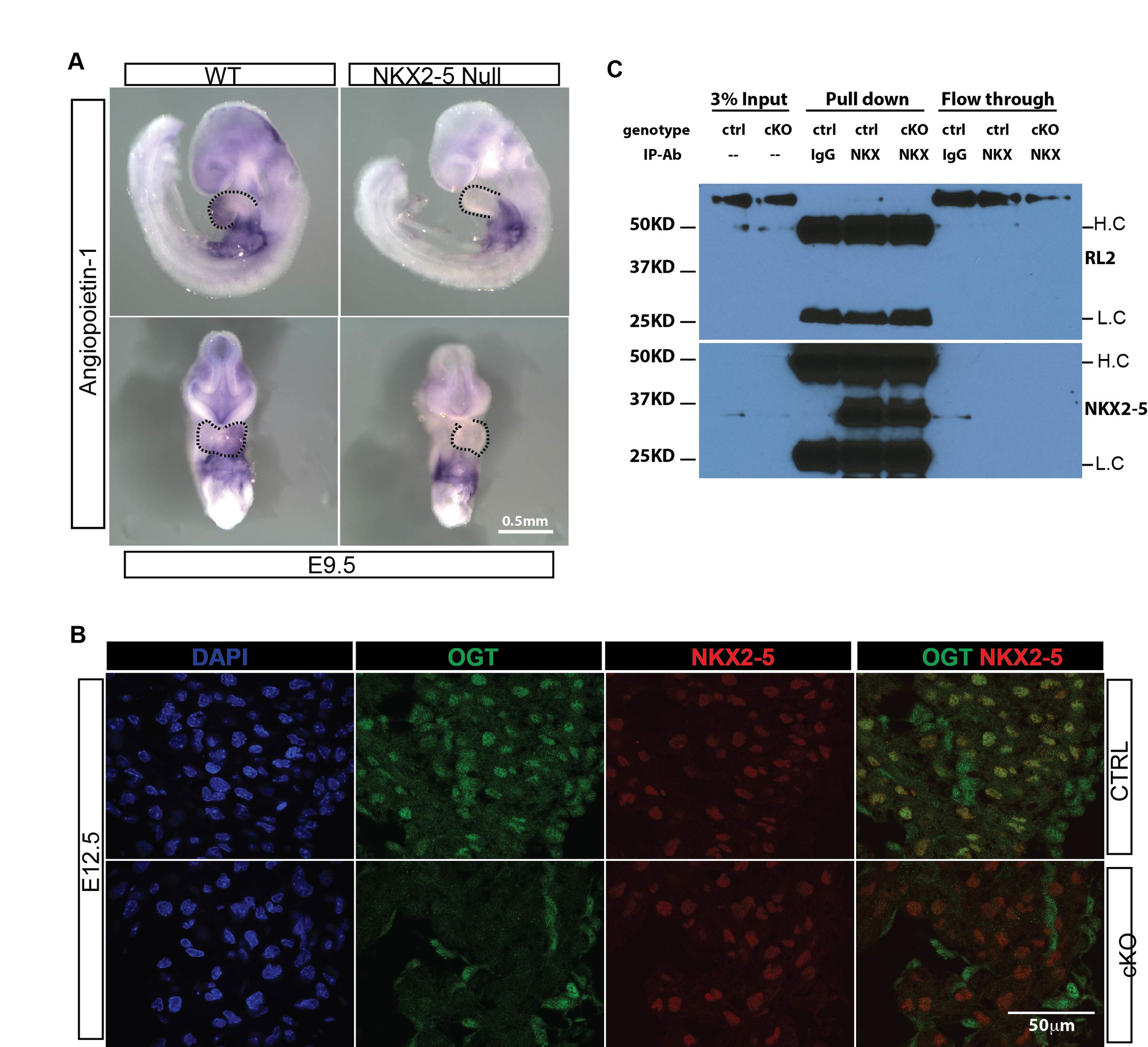

Supplement: S7 Fig — A, In situ hybridization of Angiopoietin-1 for wild type and NKX2-5 null embryos at E9.0. Heart areas are indicated by dashed lines. B, Immunoprecipitation with NKX2-5 antibody using E14.5 control and cKO heart lysate followed by immunoblotting using antibodies against O-GlcNAc (RL2) and NKX2-5 respectively. H.C., heavy chain; L.C., light chain. C, Immunostaining of cross section of E12.5 control and cKO hearts. DAPI (blue), OGT (green) and NKX2-5 (red). (TIF) [file pgen.1008730.s007.tif]
