# Supplementary material for: O-linked β-N-acetylglucosamine transferase plays an essential role in heart development through regulating angiopoietin-1
Source: PLoS Genet. 2020 Apr 6;16(4):e1008730. doi: 10.1371/journal.pgen.1008730 (PMC7182263; doi:10.1371/journal.pgen.1008730)
Supplement: S1 Table — (DOCX) [file pgen.1008730.s008.docx]

|  |  | **Genotype Distribution (number and percentage)** | | | |
| --- | --- | --- | --- | --- | --- |
|  | Total Number | *Ogt*^f/+^ | *Ogt*^f/+^, *Cre*^+^ | *Ogt*^f/y^ | *Ogt*^f/y^, *Cre*^+^ |
| E10.5 | 83 | 17 (20.5%) | 23 (27.7%) | 23 (27.7%) | 20 (24.1%) |
| E12.5 | 44 | 6 (13.6%) | 11 (25.0%) | 12 (27.3%) | 15 (34.1%) |
| E14.5 | 65 | 15 (23.1%) | 19(29.2%) | 13 (20.0%) | 18 (27.7%) |
| E16.5 | 32 | 8 (25.0%) | 5 (15.6%) | 8 (25.0%) | 11 (34.4%) |
| E18.5 | 20 | 4 (20.0%) | 5 (25.0%) | 5 (25.0%) | 6 (30.0%) |
| PD1 | 47 | 11 (23.4%) | 9 (19.1%) | 13 (27.7%) | 14*(29.8%) |
| PD21 | 100 | 36 (36%) | 27 (27%) | 37 (37.0%) | 0 (0.0%) |

*, 6 of 14 *Ogt*^f/y^, *Cre*^+^ mice were already dead at the time of genotyping.
