# Supplementary material for: O-linked β-N-acetylglucosamine transferase plays an essential role in heart development through regulating angiopoietin-1
Source: PLoS Genet. 2020 Apr 6;16(4):e1008730. doi: 10.1371/journal.pgen.1008730 (PMC7182263; doi:10.1371/journal.pgen.1008730)
Supplement: S2 Table — (DOCX) [file pgen.1008730.s009.docx]

| Gene | Forward primer (5’—3’) | Reverse primer (5’—3’) |
| --- | --- | --- |
| *18s* | GGAAGGGCACCACCAGGAGT | TGCAGCCCCGGACATCTAAG |
| *Ogt* | CTGTCACCCTTGACCCAAAT | ATGGGGTTGCAGTTCGATAG |
| *Mgea5* | CTCCTTGGTGTGCTGGATGAA | AGCTCAATGGCCTGAACCAA |
| *Gja5* | CACCCACCGTTCCTCTAAAA | AGAAGAACCCGAGAAGCACA |
| *Angpt1* | GGTGTTTTGCTAAAGGGAGGAA | TGAACTCGTTCCCAAGCCAA |
| *Mmachc* | TTGACAGAGCCCTCAAACCC | TTCAATCCCTGGCAGCAACA |
| *Rec8* | TTTCAACAGTGCCAGTACCTTG | CGGGGTTGCAGCCTCTAAAA |
| *Tnni2* | CGAAGATCGACGTGGCTGAA | AACTTGCCCCTCAGGTCAAA |
| *Tnnt3* | GACAAGGCCAAGGAACTCTG | CAGGAGTCAGGGTTGATGGT |
| *Klf15* | CCAGCAGCAGAACTTCTCAA | TTTGGAGAGGTGGTCACTCC |
| *Vcam1* | AAGGGACGATTCCGGCATTT | TCGGGCACATTTCCACAAGT |
